# Experiences of Patient-Led Surveillance, Including Patient-Performed Teledermoscopy, in the MEL-SELF Pilot Randomized Controlled Trial: Qualitative Interview Study

**DOI:** 10.2196/35916

**Published:** 2022-07-01

**Authors:** Dorothy Drabarek, Emily Habgood, Monika Janda, Jolyn Hersch, Deonna Ackermann, Don Low, Cynthia Low, Rachael L Morton, Mbathio Dieng, Anne E Cust, Adelaide Morgan, Elloise Smith, Katy L J Bell

**Affiliations:** 1 Sydney School of Public Health University of Sydney Sydney Australia; 2 Centre for Cancer Research and Department of General Practice University of Melbourne Melbourne Australia; 3 Centre for Health Services Research University of Queensland Brisbane Australia; 4 Cancer Voices NSW Sydney Australia; 5 NHMRC Clinical Trials Centre The University of Sydney Sydney Australia; 6 The Daffodil Centre The University of Sydney Cancer Council NSW Sydney Australia; 7 Melanoma Institute Australia The University of Sydney Sydney Australia

**Keywords:** melanoma, self-surveillance, digital technologies, teledermoscopy, teledermatology, mHealth, mobile phone

## Abstract

**Background:**

Current clinician-led melanoma surveillance models require frequent routinely scheduled clinic visits, with associated travel, cost, and time burden for patients. Patient-led surveillance is a new model of follow-up care that could reduce health care use such as clinic visits and medical procedures and their associated costs, increase access to care, and promote early diagnosis of a subsequent new melanoma after treatment of a primary melanoma. Understanding patient experiences may allow improvements in implementation.

**Objective:**

This study aims to explore patients’ experiences and perceptions of patient-led surveillance during the 6 months of participation in the MEL-SELF pilot randomized controlled trial. Patient-led surveillance comprised regular skin self-examination, use of a mobile dermatoscope to image lesions of concern, and a smartphone app to track and send images to a teledermatologist for review, in addition to usual care.

**Methods:**

Semistructured interviews were conducted with patients previously treated for melanoma localized to the skin in New South Wales, Australia, who were randomized to the patient-led surveillance (intervention group) in the trial. Thematic analysis was used to analyze the data with reference to the technology acceptance model.

**Results:**

We interviewed 20 patients (n=8, 40% women and n=12, 60% men; median age 62 years). Patients who were more adherent experienced benefits such as increased awareness of their skin and improved skin self-examination practice, early detection of melanomas, and opportunities to be proactive in managing their clinical follow-up. Most participants experienced difficulty in obtaining clear images and technical problems with the app. These barriers were overcome or persevered by participants with previous experience with digital technology and with effective help from a skin check partner (such as a spouse, sibling, or friend). Having too many or too few moles decreased perceived usefulness.

**Conclusions:**

Patients with melanoma are receptive to and experience benefits from patient-led surveillance using teledermoscopy. Increased provision of training and technical support to patients and their skin check partners may help to realize the full potential benefits of this new model of melanoma surveillance.

## Introduction

### Background

Globally, there is a large and growing number of people treated for melanoma localized to the skin who require ongoing surveillance for subsequent new melanoma [1]. Patients are recommended to attend routinely scheduled clinics at intervals varying between 3 and 12 months (clinician-led surveillance) to facilitate early detection of subsequent new primary or recurrent melanoma [2]. However, the optimum frequency and duration of follow-up and the clinical effectiveness of clinician-led surveillance are uncertain [3,4]. Many subsequent melanomas are detected by patients themselves, partners, or family members between scheduled visits [5,6].

These observations have led to the proposal of a new model of follow-up care called patient-led surveillance. This model involves regular and thorough skin self-examination (SSE), teledermatology facilitated monitoring, access to fast-tracked unscheduled clinic visits should the patient identify a lesion confirmed as concerning by the teledermatologist, and potentially fewer routinely scheduled clinic visits [7]. Mobile teledermoscopy is a *mobile health store and forward technology* in which patients use a mobile dermatoscope that attaches to their smartphone camera during their SSE [8]. A smartphone app is then used to process, track, and send high-quality images to a teledermatologist for assessment [9]. The adoption of mobile health technology interventions and telehealth is dependent upon their acceptance by patients and their treating clinicians [10,11]. Patients at risk of subsequent melanoma have reported that mobile teledermoscopy is acceptable when asked about its hypothetical use [9,12,13] and, in one study, after trying it out themselves (used on a one-off basis) [14].

### Objectives

The MEL-SELF pilot randomized controlled trial (RCT) [15] (ACTRN12616001716459) compared 6 months of patient-led surveillance in addition to usual care (intervention) with clinician-led surveillance (usual care; control). The intervention was found to increase SSE frequency and thoroughness, clinic visit frequency, skin lesion excision, and diagnoses of subsequent new primary melanoma ahead of routinely scheduled visits, with no detectable effect on adverse psychological outcomes. Adherence to the intervention was suboptimal, with only half of the patients submitting any images for teledermatology because of withdrawals and nonresponse. In this nested qualitative study among a subset of intervention arm participants, we aimed to explore patients’ perceptions and experiences of patient-led surveillance using mobile teledermoscopy, to understand possible determinants of adherence, and to identify opportunities for improving implementation of the intervention during the larger RCT.

## Methods

### Intervention Overview

The MEL-SELF pilot RCT was conducted from November 2018 to January 2020 and recruited 100 patients attending routine melanoma follow-up at 4 skin cancer clinics in Sydney and Newcastle, New South Wales, Australia (3 specialist-led clinics and 1 general practitioner–led clinic). Intervention arm participants were supported to undertake regular SSE and patient-performed teledermoscopy every 2 months. Teledermoscopy tools were provided by MetaOptima Technology Inc. [16], including a mobile dermatoscope (MoleScope I) that integrates with MoleScope (smartphone-based skin imaging app) [17] and DermEngine (a digital software system that facilitates the capture, storage, communication, and analysis of skin images by dermatologists) [18]. Each intervention arm participant also received a booklet of instructions and instructional videos. At the end of the 6-month study period, all 49 intervention arm participants were invited to participate in the qualitative study via postal mail and email, with follow-up invitations as needed.

### Ethics Approval

This study was approved by the University of Sydney Human Research Ethics Committee (X15-0445) and the Royal Prince Alfred Hospital (HREC/15/RPAH/593). All participants provided informed consent. The reporting of this study followed the Standards for Reporting Qualitative Research [19].

### Data Collection and Analysis

Semistructured telephonic interviews were conducted between February and March 2020. An interview guide (Multimedia Appendix 1) was developed by the authors, including the 2 consumer investigators (CL and DL). The interviews were conducted by 3 members of the research team, all trained in qualitative interviewing (EH, AM, and ES). The interviews were audio recorded and transcribed verbatim using a transcription service. Quantitative data on demographic and clinical characteristics and on adherence were collected as part of the pilot RCT using web-based surveys (REDCap [Research Electronic Data Capture; Vanderbilt University]) and the data analytics on image submission from the trial’s teledermatology platform (DermEngine). RCT data regarding occupation were clarified and expanded upon in the qualitative interviews and then used to categorize the participants into occupation groups. Adherence data from the pilot RCT were used to group participants into categories of adherence, and then, we compared patient accounts between and within these categories. Preliminary codes were developed inductively from a subset of 6 transcripts [20] independently by 2 researchers, both experienced in thematic analysis (EH and DD). Preliminary codes and analytic memos were reviewed by the research team, which included researchers from a range of backgrounds, including clinical epidemiology, health psychology, behavioral science, and health economics. The emerging themes were identified as analogous to the constructs of the technology acceptance model (TAM). The general TAM framework posits that a person’s *intent to use* and *actual use* are predicated on their perception of the technology’s *ease of use* (usability) and *usefulness* (benefit) [21] and has been used previously to assess the acceptability of apps to support health care delivery [22-29]. Thereafter, we used an inductive and deductive coding approach based on TAM [30]. Agreement between coders (DD and EH) was high, and discrepancies were resolved through consensus. The framework analytic method was used to organize codes, identify themes, and explain how they relate to each other [31]. Data saturation and interpretation were determined through ongoing coding of the remaining transcripts and discussions with the research team. Coding was performed in Microsoft Word, and Microsoft Excel was used for the thematic analysis using a data matrix.

## Results

### Overview

**Figure 1:**
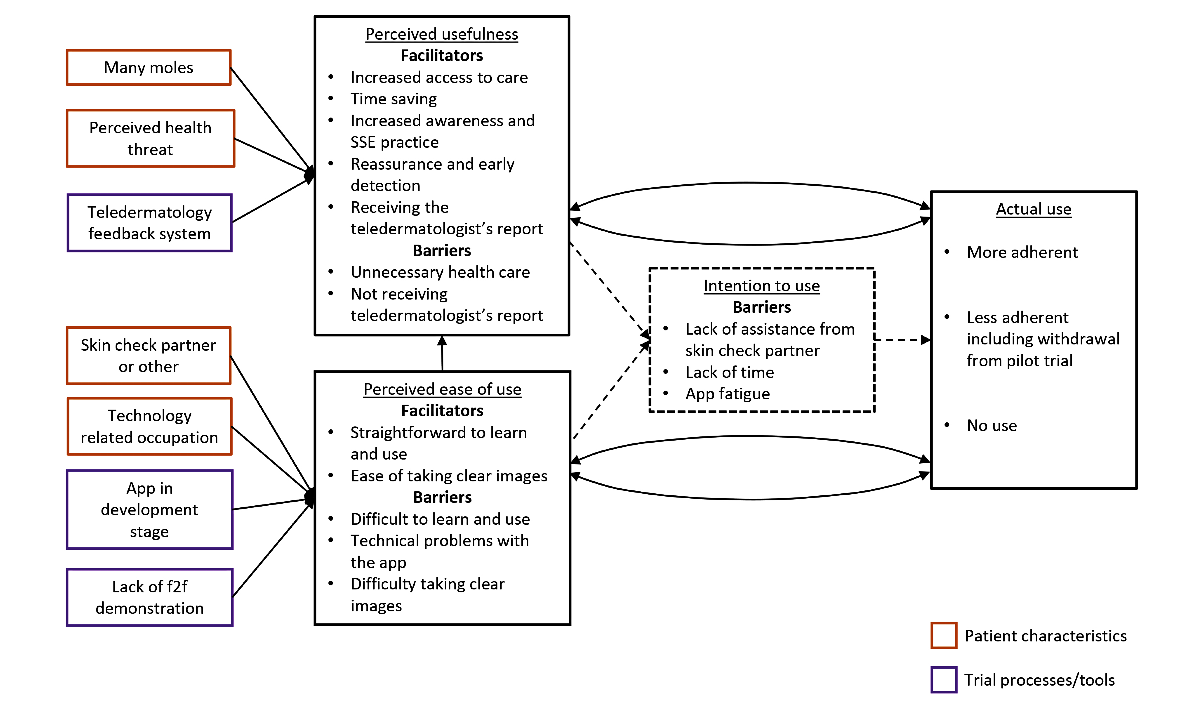
Adjusted and extended technology acceptance model for MEL-SELF pilot trial. f2f: face-to-face; SSE: skin self-examination.

Of the 49 intervention arm participants invited, 43 (88%) responded and 20 (41%) agreed to participate in a telephone interview. Interviews ranged in duration from 13 to 36 minutes. Those who participated in an interview were more likely to have submitted at least one image (16/20, 80%) compared with 53% (26/49) of intervention arm participants. Participants’ demographics and frequency of image submission are summarized in Table 1. All interviewees thought that the intervention was a *useful concept* and a *great idea* for people treated for melanoma. They said that it could potentially provide quick access to an expert’s opinion between scheduled visits and save time on physician’s appointments, particularly for those who live at a distance from specialist services and would otherwise delay accessing care. We interpreted these hypothetical benefits (motivation to use the intervention) and reasons participants gave for not using the intervention at all as *intention to use*. Patterns of use throughout the trial follow-up (including no use) were interpreted as *actual use*. Figure 1 shows the adapted and extended TAM. Multimedia Appendix 2 includes additional illustrative quotes.

**Table 1:** Characteristics of qualitative study and total intervention arm participants.

Characteristics		Qualitative study participants (N=20)	Total intervention arm participants (N=49)^a^
**Sex^b^, n (%)**			
	Male	12 (60)	26 (54)
	Female	8 (40)	22 (46)
Age (years), mean (SD; range)		57.4 (13.2; 28-78)	57.5 (12.3; 28-78)
**Remoteness area (by postcode) [32]^c^, n (%)**			
	Major cities (metro)	17 (89)	38 (88)
	Inner regional (regional)	2 (11)	5 (12)
**AJCC^d^ melanoma substage of first primary melanoma, n (%)**			
	0	7 (35)	18 (38)
	IA	12 (60)	27 (56)
	IB	1 (5)	3 (6)
Digital technology–related occupation (yes)^e,f^, n (%)		5 (25)	5 (10)
Time since first diagnosis (years), median (range)		4.7 (0.1-20.7)	5.5 (0.1-41.2)
**Frequency of image submission (time points)^g^, n (%)**			
	0	4 (20)	23 (47)
	1	6 (30)	12 (25)
	2	9 (45)	12 (25)
	3	1 (5)	2 (4)
Total number of images submitted, median (range)		6.5 (0-32)	2 (0-35)
Melanomas detected at nonscheduled visits, n (%)		2 (10)	5 (10)

^a^Percentages may not add to 100% because of rounding.

^b^Missing data for 1 intervention arm participant.

^c^Missing data for 1 qualitative study participant and 6 intervention arm participants.

^d^AJCC: American Joint Committee on Cancer, 8th Edition.

^e^An occupation was considered digital technology–related when primary work tasks involved working with apps, using advanced or programing software, or in information technology. *Retired* was counted as *no*.

^f^Data missing for 1 qualitative study participant and 7 intervention arm participants.

^g^Frequency refers to image submissions where there was at least a 1-month interval between submissions. A total of 3 submissions indicated that images were submitted at all 3 time points (2, 4, and 6 months).

### Actual Use

Of the 20 intervention arm participants interviewed, 16 (80%) used the intervention tools to image lesions and submit them to a teledermatologist for review. Of these 16 participants, 1 (6%) submitted images at all 3 time points and 9 (56%) submitted images at 2 time points. These 10 participants (10/20, 50% of interviewees) were categorized as *more adherent*. Of the 20 participants, 6 (30%) submitted images at 1 time point; they are referred to as *less adherent*. The remaining (4/20, 20%) participants did not submit any images and were referred to as *nonadherent*. These 4 participants provided important data to help understand the high rate of nonadherence in the trial. One participant did not have a compatible smartphone, and 2 participants did not use the tools because of competing time commitments and the unavailability of their skin check partner. The fourth participant reported a sense of *tech overload* or *app fatigue* and did not want to use yet another app. All 4 participants believed that their existing routinely scheduled clinic follow-up visits were sufficient for melanoma surveillance.

### Perceived Ease of Use

#### Skin Check Partner or Other Helper

Having a skin check partner was an eligibility criterion for participation in the pilot trial. Among the qualitative study sample, skin check partners included spouses, friends, or siblings. They were especially helpful for imaging difficult-to-view areas such as the back or back of the legs or when the lesion was on the participant’s dominant hand or arm. All participants who reported helpful assistance from a skin check partner were among the more adherent, and some participants reflected that it would not be possible to use the intervention successfully without help. Apprehension about using the technologies, mentioned by the 2 oldest patients aged 78 years and 73 years, was mitigated in both cases by having a *tech savvy* family member or skin check partner to assist:

I mean the technology is quite a bit new to us because we’re in the older generation. But my grandson helped me get it going...so that worked out okay.

male, 78 years, regional, nondigital technology occupation

However, having a nominated skin check partner did not always mean that they were available or were able to provide effective assistance. One participant reported that their skin check partner was reluctant to be involved and did not provide any help. Despite this, she persevered and submitted images at 2 time points, but she found the process difficult. Among those less adherent, only 1 participant mentioned having a helpful partner, and there was generally little mention of working together with someone to take images.

#### Digital Technology–Related Occupation

Participants who had the least amount of trouble learning to use the intervention tools and found the written instructions adequate tended to work in digital technology–related occupations. These participants were among the more adherent. When these participants encountered developmental glitches and *bugs* in the app (such as when indicating a newly identified mole on the full-body view or uploading and submitting images), they were able to recognize that these problems were most likely because of the app, rather than lack of their skill or knowledge. One participant said of the intervention that *the pros outweigh the cons* and subsequently persevered through problems with the app. Among participants who were less adherent, none worked in digital technology–related occupations and tended to experience more frustration and uncertainty when they could not use or navigate the app with ease.

#### Taking Clear Images

Participants and their skin check partners were conscious of the importance of taking images of adequate quality. Participants who were more adherent tended to report that the dermatoscope was easy to use or did not mention any problems using the dermatoscope, whereas those who adhered less tended to be less confident. For them and their skin check partner, taking a good quality image became stressful and time consuming, involving *fiddling around* and taking several images before uploading one that they thought was of adequate quality. Some were unsure of the correct technique in terms of knowing the right amount of fluid or how much pressure to apply when holding the device against the skin.

#### Lack of Face-to-face Demonstration

The remaining 3 participants said that the instructions provided were adequate. Each of these 3 participants had a digital technology–related occupation. Of the 20 participants, 17 (85%) said that face-to-face training and demonstration would have been beneficial to make the process of learning to use a new technology more efficient and to increase their confidence in whether they were doing the *right thing*:

It would have made things easier. For instance, if there was a session where everyone was handed their little lens for the phone and to just have a practice and be told which part of the app to go to in which sequence for instance.

female, 60 years, metro, nondigital technology occupation

Participants also said that face-to-face instruction would have provided an opportunity to ask for clarification of trial instructions such as how many moles to image per time point and an explanation of where the images were sent. Importantly, participants mentioned that because skin check partners were taking the images, they should be included in any demonstration and training:

My wife wasn’t sure exactly...and I couldn’t help her by not seeing where she was photographing...I couldn’t know if she was doing it right or wrong, but I think it would’ve been easier for both of us to go up there and just get a demonstration, to make sure we did it right.

male, 63 years, Metro, nondigital technology occupation

There was also a general sense of having to *get used to* the intervention, in terms of using the tools with confidence and integrating them into an SSE routine.

### Perceived Usefulness

#### Increased Awareness and SSE Practice

Among those who had least difficulty in using the intervention and were more adherent, there was a strong sense of increased awareness of what was happening on their skin. This included looking more closely at moles that they would not have looked at otherwise and conducting more regular skin checks:

So, it gave me an opportunity—like it gives you a regime and it puts a tool in your hands, so it means that you pay more attention.

male, 53 years, metro, digital technology–related occupation

Another participant felt empowered by being more involved in their melanoma follow-up:

So going on the trial was good because I could get that extra sense of control over what was happening with my moles. I could be watching it more carefully...it was an extra chance to be proactive.

female, 43 years, metro, digital technology–related occupation

Another participant appreciated having easy access to a *track record* of concerning moles on their phone. For 2 participants, increased awareness of their skin caused additional anxiety because of the possibility of finding another melanoma; however, this did not detract from their perception of the usefulness of the intervention or impact their adherence.

#### Reassurance and Early Detection

Participants who were more adherent explained that they felt reassured with having access to additional care, meaning that concerning lesions were being monitored between scheduled appointments and, if necessary, action would be taken:

Well, you know that someone is checking on you monthly. So, to me, that’s a good thing...And if they say to come back and get your doctor to check, well, really that’s only for your benefit, isn’t it?

female, 60 years, regional, nondigital technology occupation

A total of 2 participants, also among those who were more adherent, highlighted their experience of the intervention’s ability to facilitate the early detection of melanoma. They reported having a melanoma detected and diagnosed 2 months and 3 months ahead of their next routinely scheduled appointment:

Well, it picked up a melanoma, I thought that was just amazing, otherwise, it would have been another three months before they picked it up.

male, 73 years, metro, unknown occupation

#### Having Many Moles

For the 4 participants who reported having dysplastic nevus syndrome, high perceived health threat influenced the stress and anxiety associated with the intervention. Three participants suggested that it may be more useful for people with fewer moles. Stress was caused by the possibility of not identifying moles of concern, not knowing which moles to image, having trouble finding the same mole that they had previously imaged, and having to arrange for additional clinic visits. These concerns were shared by the patients’ skin check partners, who were tasked with ensuring that the images were of adequate quality. Of the 4 participants, 2 (50%) stopped using the intervention after submitting images at one time point, preferring to leave the responsibility of their skin examination solely to their physician:

It’s stressful when somebody’s asking, “What about this one? What about that one?” and at the end of the day, if you’re spending too much time it becomes, “Did we miss one?” or “Should we have put that one on?”...for me, I’d rather have him checking it every three months because of what I’ve been through for the past four or five years, you know.

male, 69 years, metro, nondigital technology occupation

Of these 2 participants, 1 (50%) also found the tools very difficult to use, which compounded his frustration causing him to “chuck it [the intervention] in the too hard basket.” The other 2 participants were among those who were more adherent. One participant reported that they would definitely keep using digital technologies beyond the trial if given the opportunity, and the other participant said that they would be unlikely to do so.

A total of 2 other participants said that confidence in using the intervention may increase if the moles to image were chosen with the physician, thereby reducing reliance on the participant’s or skin check partner’s ability to discern which moles were of most concern:

...maybe selecting the spots on your body in consultation with your doctor would make you feel more confident.

female, 60 years, metro, nondigital technology occupation

#### Unnecessary Health Care Use

A total of 3 participants reported that the intervention resulted in unnecessary care. For 1 patient with many moles, the intervention prompted several additional clinic visits for lesions that were already being monitored by the treating physician. This caused the patient to question the usefulness of the additional visits, that had resulted in quite a lot of anxiety and an increase in health care costs. The need to image a prescribed minimum number of concerning moles also caused anxiety for this patient, as each additional mole photographed was potentially another skin cancer:

I’ve already had anxiety, but every time I submitted my pictures, I was told I had to find three or four—there was a requirement for moles that I needed to note if I detected changes or wanted to be monitored and obviously, you’re like, “I guess I’ve got to find another one.”

female, 43 years, metro, digital technology–related occupation

Another participant, also with many moles, recounted that having to look for a prescribed number of new concerning moles was not helpful, as he did not know which ones to choose from. In addition, in the fast-tracked appointment that he was requested to make, it turned out that an image of a stretched lesion in which too much pressure had been applied had prompted the recommendation. A third participant, who had very few moles, felt that the intervention was not useful for them because they felt compelled to submit images of lesions they were not concerned about. This participant was among those who were less adherent. It is important to highlight these instances of unnecessary care; however, they did not have a clear effect on adherence.

#### Receiving the Teledermatologist’s Report

Feedback from a teledermatologist was received successfully by some participants, one saying that the response time as *excellent*. However, for others, the lack of timely feedback from the teledermatologist put the usefulness of the intervention into question. Technical problems with the teledermatology feedback loop including absence of a *sent* confirmation or not receiving the teledermatologist’s report at all resulted in uncertainty. Participants explained that they were not sure if they had used the intervention correctly, if the images had been received at the other end, or if they were required to make an appointment with their physician:

...this happened a couple of times, when I submitted something that I felt was unusual...nothing came back, I didn’t get a response, I didn’t get a report from the specialist on the receiving end...So, I think there was a little bit of a disconnect initially...

male, 53 years, metro, digital technology–related occupation

Receiving the teledermatologist’s report also caused anxiety for some participants, but not more anxiety than they experienced when receiving other test results about their melanoma risk. The use of the word *urgent* in the teledermatologist report provoked some alarm, but this was balanced by the reassurance of knowing that if another melanoma was suspected, then action could be taken. Receiving the teledermatologist’s report is integral to the usefulness of the intervention; however, whether receiving feedback had a clear impact on adherence is difficult to discern, as most accounts of problems with feedback came from those who were more adherent and for whom there were more opportunities for problems to occur.

## Discussion

### Principal Findings

In this qualitative study of patients randomized to patient-led melanoma surveillance using teledermoscopy, we found that, in practice, among participants who were more adherent (submitted images at 2 or 3 time points), the intervention prompted increased awareness of their skin and SSE practice, reassurance, and early detection of subsequent melanoma. These more adherent participants were those who found the intervention easier to use because of working in a digital technology–related occupation and by having an effective skin check partner. Those who submitted images at only 1 time point found the tools too difficult to use. This outweighed the perceived potential benefits and impacted their intention to use the intervention tools. Although a few participants found the intervention tools easy to use from the start, most participants experienced varying degrees of difficulty in taking clear images and encountered several developmental glitches and navigational issues in the app. These participants needed repeated practice before they found the tools easier to use. Perceived usefulness was lower in people with many moles, especially when it prompted unnecessary clinic visits. Those who did not submit any images explained that their nonadherence was because of competing time commitments, not having an available skin check partner, not having a compatible mobile phone, or *app fatigue*.

In our study, anxiety was not a clear delineating factor between those who were more or less adherent; however, it was present in the experiences of most participants and their skin check partners. High perceived health threat associated with a personal history of melanoma, increased patient and skin check partner anxiety, particularly among those with many moles. Accounts of stress and anxiety among these patients in our study and in other studies suggest that patients with many moles may require more ongoing support to conduct patient-led melanoma surveillance because of difficulty in selecting moles and increased risk [14,33].

Aligning with the core hypotheses of the TAM framework [21], we found that ease of use and usefulness influenced intent to use and actual use. However, we also found that despite the initial intention to use, perceptions of ease of use and usefulness after the follow-up period were more influential in explaining actual use. Our findings also indicate that actual use impacted ease of use and usefulness, in that those who used the intervention over a longer period found it easier to use (after getting used to it) and experienced more of its benefits. TAM is commonly used to assess factors that influence the intention to use health technologies. Intention to use is interpreted as a measure of acceptability [23,25,27], even when participants are only asked about hypothetical use [26] or it is not clear if all participants have used the technology [29]. In studies that include actual use in their final TAM model, intention to use is not always a strong or statistically significant predictor of actual use [24,25]. Intention to use, measured hypothetically or after a short period of use, may not always be a good predictor of actual use behavior [28], particularly when digital technology requires a period of learning and is being used in the management of high-risk conditions. Qualitative assessment *after* implementation, and over time, may produce a more accurate and comprehensive understanding of context and patient and intervention characteristics that influence actual use behavior [34] to better inform implementation strategies.

Overwhelmingly, participants suggested that training and demonstration were necessary for themselves and their skin check partner. One-to-one training in SSE has been found to result in greater SSE skill acquisition compared with a paper workbook or electronic interactive training [35]. Previous research has found that a partner’s attendance at SSE skills training increased the frequency of SSE [36,37]. A patient and partner working together as a *dyad* has also been found to improve SSE practice [38]. In their assessment of mobile teledermoscopy, Horsham et al [14] found that most participants had the help of a family member to take photos and submit images. However, the necessity of having a skin check partner excludes people who do not have someone to help them take images regularly, and further consideration is needed on how to best support these people to undertake regular self-surveillance and act on their results.

The lack of image submission by almost half of all intervention participants in the pilot RCT could be explained by the additional effort and time needed to learn to use the intervention and then use it routinely with a skin check partner, in addition to usual care. When combined with a high perceived health threat from their increased melanoma risk, this may mean that some patients prefer to rely solely on their physician for follow-up care [7,34]. However, early one-on-one training and demonstration may make the learning process less daunting, encourage participation of skin check partners, and create a supportive connection between trial staff and participants. This may encourage more participants to try the intervention and to continue to engage with it over a longer term. Among our study sample, those who used the intervention over a longer period reported more positively on ease of use and usefulness, highlighting the importance of supporting skill acquisition to increase self-efficacy.

Our findings have assisted in refining the design and implementation of a larger ongoing RCT on patient-led melanoma surveillance [39], and several changes have been made. To help overcome barriers to perceived usefulness, particularly for those with many moles, a *target lesion* will be selected by the treating clinician, an approach suggested in previous studies [9,40]. To reduce the potential for medical overuse [41], the need for a minimum number of lesions has been removed. If the patient does not have other lesions of concern, they will not need to submit any images other than those of the target lesion. Clinical practice guidelines consistently recommend that patients should be taught SSE, but the optimum frequency of SSE and teledermatology remains ill-defined [42]. The frequency of image submission requested in the pilot trial was assessed to be too high, as only a small proportion of participants in the pilot RCT, including only 1 qualitative study participant, were able to submit images at all 3 time points [15]. The frequency of image submission will be reduced from every 2 months to every 3 months. All intervention arm participants and their skin check partners will be encouraged to participate in one-to-one demonstration sessions with the study staff, in addition to receiving instructional videos and written instructions. The study staff will also be available for the duration of the follow-up period to assist patients with troubleshooting. In addition, the technology provider has made several improvements to the app and teledermatology feedback system, which addresses the technical difficulties experienced by the participants, including nonreceipt of the teledermatologist’s report.

The patient-led surveillance approach has the potential to partially (or completely) replace routinely scheduled visits. However, during this initial stage in which we are evaluating the safety and effectiveness of the intervention, we have implemented it in addition to usual care, both in the pilot trial that this study relates to and the larger trial that is ongoing. In the process of co-designing these studies, it was clear that both clinicians [43] and patients [12] preferred implementation in addition to usual care as a first step before considering circumstances in which it might replace (in part or in whole) routinely scheduled visits. Although we implemented the intervention in addition to usual care, we are surveying patients regarding their acceptance of a hypothetical reduction to their routinely scheduled clinic visits. We envision that this may inform situations in which the intervention might replace some routinely scheduled visits. Data from interviews with clinicians involved in the pilot trial were also informative. The clinicians indicated that after experiencing the actual use of the intervention in the pilot trial, they anticipated that in some clinical scenarios, it may replace routinely scheduled visits—in particular, where a specific lesion is being monitored for change (these findings have recently been corroborated; Drabarek, D, unpublished data, May 2022). In other scenarios, it may be used in addition to usual care to triage which patient concerns warrant review in the clinic—in particular, where a new lesion needs evaluation. Further exploration of these different uses of patient-led surveillance, the patients most likely to benefit from this approach, and integration with other approaches to surveillance [44] could help to define how it may be used in the most clinically effective and cost-effective way.

### Strengths and Limitations

As our findings draw on patient experiences over the 6-month trial period, they provide novel insights into the implementation of patient-performed teledermoscopy interventions. As the study period allowed for repeated use of the intervention tools, we were able to interrogate a variety of adherence patterns and identify facilitators and barriers to these and their determinants. These findings have assisted in anticipating and mitigating the risk factors for low adherence in the larger MEL-SELF trial and may also be useful for future studies. Our findings also draw on the experiences of a study sample with variations in relevant demographic characteristics such as age, residing in metropolitan or regional areas, and digital technology self-efficacy. However, as an opt-in recruitment method was used, the qualitative substudy sample was much more adherent than those who did not agree to participate in an interview; thus, additional explanations for nonadherence may have been missed. Further research is necessary to understand the low uptake of mobile teledermoscopy interventions among patients. In addition, some aspects of the results reflect the use of software that was in its development phase, and the findings may not be transferrable to patients using teledermoscopy technologies created by different developers. A time frame longer than 6 months may have revealed further determinants of actual use. Finally, because interviews were conducted at the end of the pilot trial, experiences of learning to use the intervention tools at the beginning of the trial may not always have been reported accurately.

### Conclusions

Patient-led surveillance is a complex behavioral change intervention. It aims to improve patients’ knowledge, skills, and confidence in performing SSE using digital technologies so that they are better able to detect and act on concerning changes to moles and other skin lesions. Understanding how and why patients do or do not use this intervention is fundamental to increasing adherence within a clinical trial setting and increasing uptake in clinical practice, if it is found to be a clinically beneficial and cost-effective method of melanoma surveillance. Ultimately, it may allow access to melanoma follow-up care regardless of geographical location [45] and could become a *new normal* method of surveillance after the COVID-19 pandemic [46].

## Appendix

Multimedia Appendix 1 Interview topic guide.

Multimedia Appendix 2 Illustrative quotations.

## Abbreviations

NHMRC: National Health and Medical Research Council
RCT: randomized controlled trial
REDCap: Research Electronic Data Capture
SSE: skin self-examination
TAM: technology acceptance model
