# Supplementary material for: Experiences of Patient-Led Surveillance, Including Patient-Performed Teledermoscopy, in the MEL-SELF Pilot Randomized Controlled Trial: Qualitative Interview Study
Source: JMIR Dermatol. 2022 Jul 1;5(3):e35916. doi: 10.2196/35916 (PMC10334928; doi:10.2196/35916)
Supplement: Multimedia Appendix 1 [file derma_v5i3e35916_app1.docx]

**Appendix 1: Interview topic guide**

**Opening questions:**

1. Can you tell me when your melanoma was diagnosed and how it was detected?

**Experience with MEL-SELF**

1. Moving now to the study that you’ve participated in over the past six months in which you were given an attachment for your phone and an app to send photos in, overall, how would you describe your experience/opinion of “MEL-SELF”?
   - Prompt: Did you take and submit any photos?
   - Prompts if withdrawn: Can you tell me about why you stopped participating in the study? What would have made it easier for you to continue with the study?
2. Can you tell me about the training or instructions you received prior to commencing the trial?

- Prompts: Was the training helpful? What parts did you like/Not like? What parts did you remember / forget? What could be improved? Did you have further training needs that weren’t met?
  - 1. In person by doctor or site coordinator
    2. Written instructions
    3. Video

1. Can you tell me about how you felt that the study impacted on your usual care or appointments with your doctor?
   - Prompts: did you discuss aspects of the trial in appointments with your doctor?
2. If you were going to do skin self-examination today, using the MoleScope app and dermatoscope can you describe to me the steps that you would follow?
   - Prompts: as going through steps could incorporate questions about each component (ASICA and Dermatascope/teledermatology) about acceptability and satisfaction.

**Barriers during MEL-SELF**

1. Did you find any aspects of the trial difficult /OR/ Did you find any aspects of the trial more difficult than others?
   - General prompts: Can you tell me about the difficulties that you had? What did you do when you encountered difficulties with that aspect of the trial? Did you know who to contact if you needed help with a study step? How could your experience with that aspect of the trial have been improved? Did you know who was looking after you for the study? Did you know how the study was linked with your usual doctor?
   - Prompt if dropped out: What are the main aspects of the trial that caused you to drop out (If they dropped out for trial-related reasons).

*Interviewer note: there will probably not be enough time to go into each aspect of the trial. Use prompts below to focus on the aspects of the trial that participant is most interested in speaking about (i.e. aspects that were most challenging for them)*

Prompts specific to particular aspects of trial

- - Questionnaires
  - Accessibility of online questionnaires
  - Understandability of online questionnaires
  - Purpose of questionnaire topics
  - Length of questionnaires / time taken to complete
- Diaries of doctor visits and costs
  - Ease of use: difficulties completing / difficulties remembering to use
  - Suggestions to make collection of this information easier / more detailed / less detailed / change of frequency
- ASICA Skin Checker
  - Accessibility
  - Ease of use and navigation
  - Understandability
- Dermatoscope and Teledermatology
  - Ease of use: difficulties attaching dermatoscope to phone / taking images / finding same moles for comparison / using MoleScope app / app navigation
  - Someone to help
  - Time taken to go through process
  - Confidence using dermatoscope / taking image / using MoleScope app
  - Accessibility: MoleScope app / teledermatology reports
  - Understandibility: teledermatology reports
- SMS/Email reminders
  - How did you feel about receiving reminders? (emotional response)
  - Content: was the content helpful? Personal/impersonal? Relevant to you?
  - Frequency: too often / not often enough
  - Effectiveness of reminders: did reminders prompt action?
- Support from site coordinators or other research team members
  - Did you feel supported by study staff?
  - Can you tell me about the support they provided? Can you tell me how they could have made you feel more supported?

1. Did you have any concerns about MEL-SELF (using the dermatoscope/teledermatology) prior to the trial? What were your concerns? Did your experience during the trial alleviate / exacerbate those concerns? (Note: concerns include data privacy, psychosocial concerns, costs)

**Concluding questions:**

1. From your perspective what was the best thing about using teledermatology?
2. From your perspective what was the worst thing about using teledermatology? What would you change about this aspect?
3. After participating in this trial, has your opinion on teledermatology/self-examination changed? How?
   - Prompt: Do you think teledermatology would save you making trips to visit your skin doctors?
4. If it was available to you would you continue with teledermatology? Why/why not?
5. Would you recommend it to other patients?
6. Is there anything else you’d like to add?

**Closing:**

Thank you very much for your time. We will use your feedback to develop and fine-tune the protocol for the full MEL-SELF trial.
