# Supplementary material for: Experiences of Patient-Led Surveillance, Including Patient-Performed Teledermoscopy, in the MEL-SELF Pilot Randomized Controlled Trial: Qualitative Interview Study
Source: JMIR Dermatol. 2022 Jul 1;5(3):e35916. doi: 10.2196/35916 (PMC10334928; doi:10.2196/35916)
Supplement: Multimedia Appendix 2 [file derma_v5i3e35916_app2.docx]

**Multimedia Appendix 2. Illustrative quotations.**

| **Table 1S: Perceived ease of use** | |
| --- | --- |
| **Theme** | **Illustrative quotes** |
| Skin check partner or other helper | “… if I had to do it myself, I could have done it but different parts of the body you actually can’t do. I mean there’s no way I could do my back for instance. My leg was a bit awkward because it was at the back of the leg, at the side. It was pretty awkward for me to do it but very easy for the other person to do it… He loved it. He’s a nurse and he loves it.” (M, 73 yrs, metro, occupation not known)  “I think he [husband] just didn’t want to be involved I suspect; he can be a bit of an impatient type… But I could get a neighbour to come for instance or a friend or something… it's really difficult no matter where the mole was, for example if you had a mole on the back of your right hand and you want to take a photo of that and you are right-handed, trying to use your left hand to take a photo, it was in an awkward position.” (F, 60 yrs, metro, non-digital tech occupation) |
| Digital technology related occupation | "I think the equipment has merit. I guess what I found initially was getting used to it. So just some idiosyncrasies with the actual app itself. Some of the navigation in the app I found to be a little bit buggy… there were some aspects like when you have the full-blown image and you wanna indicate where you’ve got a spot that you wanna have a photograph of. That seemed to work inconsistently. So, I think there’re possibly some improvements that could be made in the app itself." (M, 53 yrs, metro, digital tech related occupation)  "I work in software, so I’m probably just being a bit biased here. So, when taking the photo and uploading it, there were sometimes where the photos wouldn’t upload, and I’d have to go back. And then I’d send it off and I’ll get a message back just saying that they couldn’t see it, can I take some more photos of it. I was going by the instructions. And sometimes it worked and sometimes it wouldn’t. So, it was a bit hit and miss." (M, 48 yrs, regional, digital tech related occupation)  "Very difficult to send the photographs on to whoever it was going to… with the app, it was very hard to use.…when you went to forward it using the stages, go to the next stage, and on-forwarded, it just wouldn't do it. I had to go back to the initial thing, like you'd missed something out." (M, 63 yrs, metro, non-digital tech occupation) |
| Taking clear images | “… ‘cause some of that new technology is a bit – not concerning, but you just wanna be sure you’re doing the right thing and you do become a little bit apprehensive when you first use it and hope that the images taken are of the right quality." (M, 68 yrs, regional, non-digital tech occupation)  “…you take one, two, possibly three photos before you get a clear one and, therefore, it did become very time-consuming…” (M, 69 yrs, metro, non-digital tech occupation)  “I found it hard to use, I have to say. I was not very successful. I didn’t realise I had to push very hard to get a clear picture. I was doing it probably too lightly, so some of my photographs weren’t very successful and I had to keep redoing them.” (F, 57 yrs, metro, non-digital tech occupation) |
| Lack of face-to-face demonstration | “I guess this is where I think having the initial face-to-face explanation of the mole scope itself and the purpose of it, and the practical aspect of sending this off for someone to have a look at and having them return a report to you... I feel like it would be nice to have someone just run through that face-to-face with you, show you the tool, explain the process, this is what happens, let’s do an example and then off you go." (M, 53 yrs, metro, digital tech related occupation)  “I thought a briefing session for participants on how to use the app would be really good, instead of just sending it in the mail.” (M, 68 yrs, regional, non-digital tech occupation) |

| Table 2S: Perceived usefulness | |
| --- | --- |
| Theme | **Illustrative Quotes** |
| Increased awareness and SSE practice | "It allowed me to send through pictures of more moles than I would have perhaps looked at, so it encouraged me to look at many more definitely." (F, 60 yrs, metro, non-digital tech occupation)  “I enjoyed taking the photos and then sending them off. …also just seeing that little MoleScope – I used to just leave it on the coffee table. It would actually prompt me to just do self-assessments more often than not. Instead of waiting a month, you kind of just have a look and play around with it.” (M, 48 yrs, metro, digital tech related occupation)  “… anything to do with any of this stuff, it does produce anxiety because it's always an opportunity for new cancers to be discovered. I mean I have anxiety if I don't do it, and then time builds up and I get even more anxious. So, it's better to be doing it more regularly.” (F, 43 yrs, metro, digital tech related occupation) |
| Reassurance and early detection | "Basically, just giving myself a bit of comfort that I can do this self-assessment and send it off to someone who’s licensed – a practitioner who can actually have a look at it, tell whether there’s something going on, or everything’s okay. I think everyone needs something like that, a bit of reassurance.” (M, 48 yrs, metro, digital tech related occupation)  “…the fact that it picked up a spot that was changing, that I was able to see the dermatologist two months earlier than my annual review. So in my mind, I feel like that helps identify something much earlier. Two months before my normal appointment is great, I reckon." (F, 36 yrs, metro, non-digital tech occupation) |
| Having many moles | "So if I got five moles and like constellation, you pick the one that you think is the one that you’re monitoring but you’ve got to collectively compare it with the last image to see if it actually is the right one. So it’s a hit and miss for someone, that’s not in the profession." (M, 54 yrs, metro, digital tech related occupation)  " …that app, you’d go onto it and a little red dot would come up where you wanted to do it. But in a vicinity where that red dot is, I might have four of five of me. It was hard to pinpoint in that respect… My purpose, I feel it was easier to get the six-monthly thing and let someone take a look all over me." (M, 60 yrs, regional, non-digital tech occupation) |
| Unnecessary healthcare use | "Particularly in the end, I was told, quite a lot of them, I needed to follow up with a clinician, so I was doing a lot of trips to the doctors …I didn't feel were necessary, even though I tend to try to err on the side of caution. As I said, there was just a couple where I just I couldn't see why I was having to make the trip and, of course every trip to the doctor’s, it's like $90 or something out of pocket. It just all adds up." (F, 43 yrs, metro, digital tech related occupation)  “I was just looking for moles because you want several moles photographed… I really have one or two spots I was just keeping an eye on them… so I was struggling just sending the same thing. …I think I probably shouldn’t been given the equipment for my phone mainly because I don’t have any moles.” (F, 57 yrs, metro, non-digital tech occupation) |
| The teledermatologist’s report | “… we didn’t know that they weren’t good photos until it came into the review and the doctor said, “Yeah, that’s not a very good photo. Look, there’s not enough light or it’s too stretched or there’s not enough fluid.” (M, 54 yrs, metro, digital tech related occupation)  “Like any doctor’s report, you go, “Oh, heavens, this is another –” in this case, is it that of melanoma, but once I went to see him [doctor] and we discussed what we thought, and he just took the action then of taking biopsy. " (F, 60 yrs, regional, non-digital tech occupation)  “I wasn't confident at all about the sending off because you’d press the button, and nothing happened.” (M, 63 yrs, metro, non-digital tech occupation) |
